# Crystal structure of *cis*-1-phenyl-8-(pyridin-2-ylmeth­yl)dibenzo[1,2-*c*:2,1-*h*]-2,14-dioxa-8-aza-1-borabi­cyclo­[4.4.0]deca-3,8-diene

**DOI:** 10.1107/S2056989017016553

**Published:** 2017-11-21

**Authors:** Gabriela Ledesma, Sandra Signorella, Davi Back, Ernesto Schulz Lang

**Affiliations:** a IQUIR (Instituto de Química Rosario), Consejo Nacional de Investigaciones Científicas y Técnicas (CONICET), Facultad de Ciencias Bioquímicas y Farmacéuticas, Universidad Nacional de Rosario, Suipacha 531, (S2002LRK), Rosario, Argentina; b Laboratório de Materiais Inorgânicos, Department of Chemistry, Federal University of Santa Maria, UFSM, 97115-900 Santa Maria, RS, Brazil

**Keywords:** crystal structure, *B*-phenyl­dioxaza­borocine, N—B dative bond, zwitterionic heterocycle, C—H⋯O inter­actions

## Abstract

The title compound, C_26_H_23_BN_2_O_2_, was obtained as by product during synthetic attempts of a complexation reaction between the tripodal ligand H_2_ *L* [*N*,*N*-bis­(2-hy­droxy­benz­yl)(pyridin-2-yl)methyl­amine] and manganese(III) acetate in the presence of NaBPh_4_. The isolated *B*-phenyl dioxaza­borocine contains an N→B dative bond with a *cis* conformation. In the crystal, C—H⋯O hydrogen bonds define chains parallel to the *b-*axis direction. A comparative analysis with other structurally related derivatives is also included, together with a rationalization of the unexpected production of this zwitterionic heterocycle.

## Abstract 2

The present work describes the synthesis and crystal structure of the new *B*-phenyl­oxaza­borocine, C_26_H_23_BN_2_O_2_. The title compound adopts a zwitterionic form with a significant intra­molecular N→B dative bond and inter­molecular C—H⋯O inter­actions connecting mol­ecules parallel to the *b* axis.

## Chemical context   

As part of our research program directed at obtaining manganese complexes as bio-inspired mimetics with different nuclearity and properties (Ledesma *et al.*, 2014 ▸, 2015 ▸), we were inter­ested in coordination reactions of the tripodal tetra­dentate ligand H_2_ *L*, namely *N,N*-bis­(2-hy­droxy­benz­yl)(pyrid-2-­yl)methyl­amine. We envisaged a systematic study comprising the use of several metal-to-ligand ratios, with the idea of varying the nuclearity of the resulting compounds. Unexpectedly, however, during consecutive attempts to obtain manganese complexes derived from H_2_ *L*, we isolated the *B*-phenyl dioxaza­borocine derivative, **I**. Here we report its synthesis and crystal structure and, in order to unravel its presence, we rationalize its production under the employed reaction conditions. A comparative analysis of its structural data with that of other dioxaza­borocines is also presented.
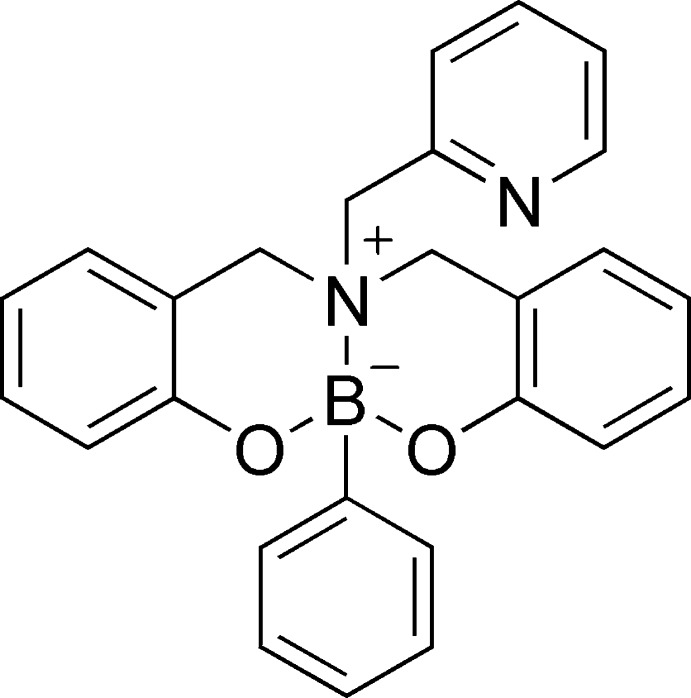


## Structural commentary   

**Figure 1:**
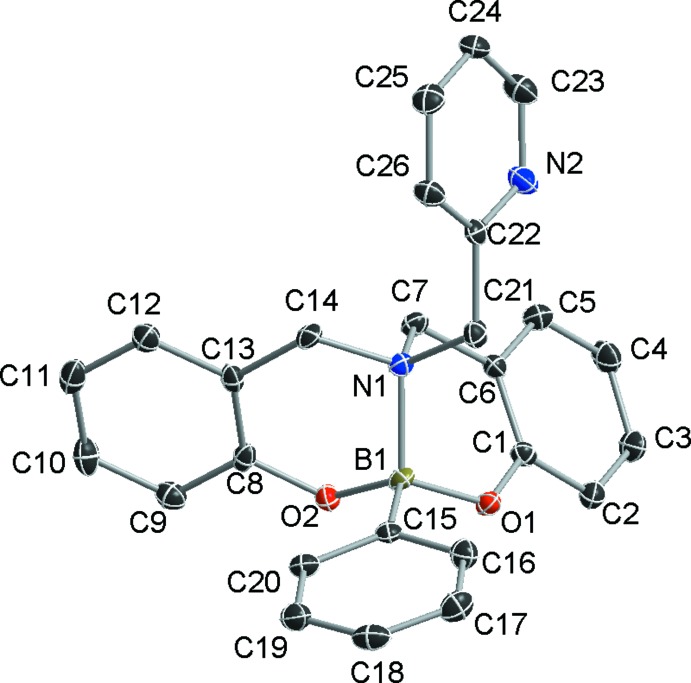
The mol­ecular structure of the title mol­ecule, with displacement ellipsoids drawn at the 50% probability level. Hydrogen atoms are omitted for clarity.

The title compound **I**, Fig. 1 ▸, which represents one of the few examples of *B*-phenyl dioxaza­borocine derivatives reported in the literature, crystallizes in the triclinic space group *P* 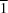 with two mol­ecules in the unit cell. The boron atom shows a distorted tetra­hedral coordination sphere described by one N atom (N1), two oxygen atoms (O1, O2) and one carbon atom from a phenyl ring (C15). The geometry about the intra­molecular N1—B1 bond is *cis*, as inferred from the spatial arrangement of atoms C15–B1–N1–C21. The B—O bond lengths are 1.446 (3) and 1.471 (3) Å and the B—N bond length is 1.674 (4) Å. The BNC_3_O six-membered rings adopt a half-chair conformation, with puckering parameters *Q*_T_ = 0.502 (2) Å, θ_2_ = 135.4 (2)°, φ_2_ = −138.4 (4)° for B1/N1/C7/C6/C1/O1, and *Q*_T_ = 0.525 (2) Å, θ_2_ = 132.2 (3)°, φ_2_ = −144.8 (4)° for B1/N1/C14/C13/C8/O2.

## Supra­molecular features   

**Figure 2:**
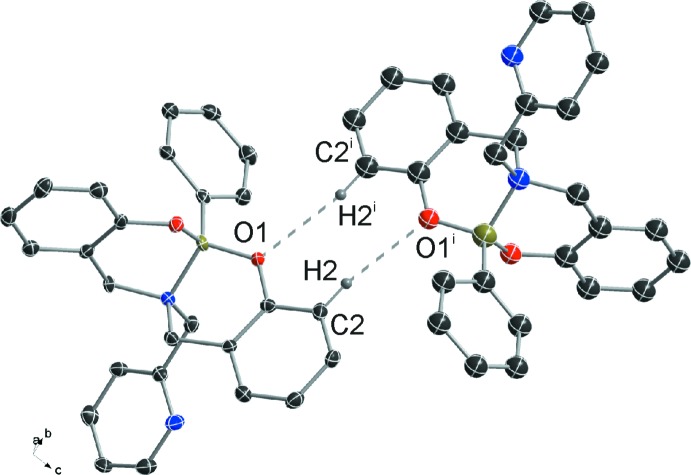
Detail of the inter­molecular inter­actions in the title compound forming a dimeric system through C—H⋯O hydrogen bonds (dashed lines). H atoms not involved in these hydrogen bonds are omitted [symmetry code: (i)= 1 − *x*, 2 − *y*, 2 − *z*].

**Figure 3:**
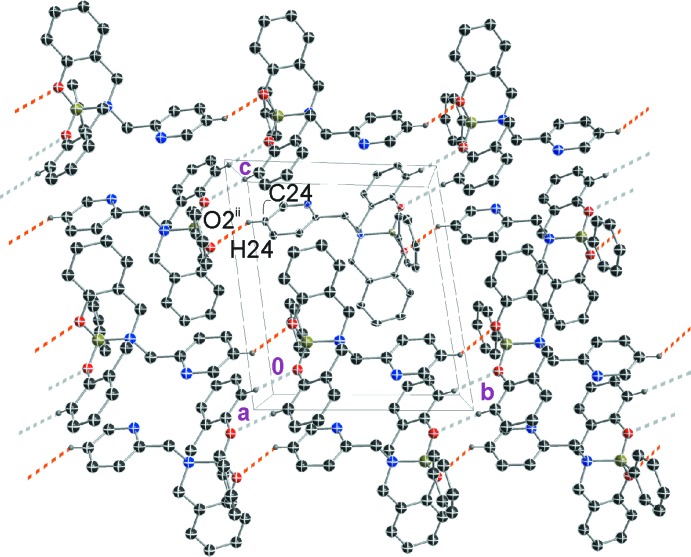
Detail of C—H⋯O inter­actions in the title compound (grey and orange dashed lines denote C2—H2⋯O1^i^ and C24—H24⋯O2^ii^ inter­actions, respectively). H atoms not involved in these inter­actions are omitted for clarity [symmetry codes: (i) 1 − *x*, 2 − *y*, 2 − *z*; (ii) *x*, −1 + *y*, *z*].

The crystal packing in **I** is defined by two sets of C—H⋯O hydrogen bonds. The first group implicates C2—H2⋯O1^i^ atoms, giving rise to a dimeric system with a C—H⋯O angles of 167.5° (Fig. 2 ▸, Table 1 ▸). The remaining inter­action, C24—H24⋯O2^ii^, shows a small C—H⋯O angle of 129.4°, indicating that this C—H⋯O hydrogen bond is quite weak. The two inter­actions link mol­ecules into chains parallel to the *b* axis (Fig. 3 ▸), consolidating the three-dimensional mol­ecular packing.

## Database survey   

**Figure 4:**
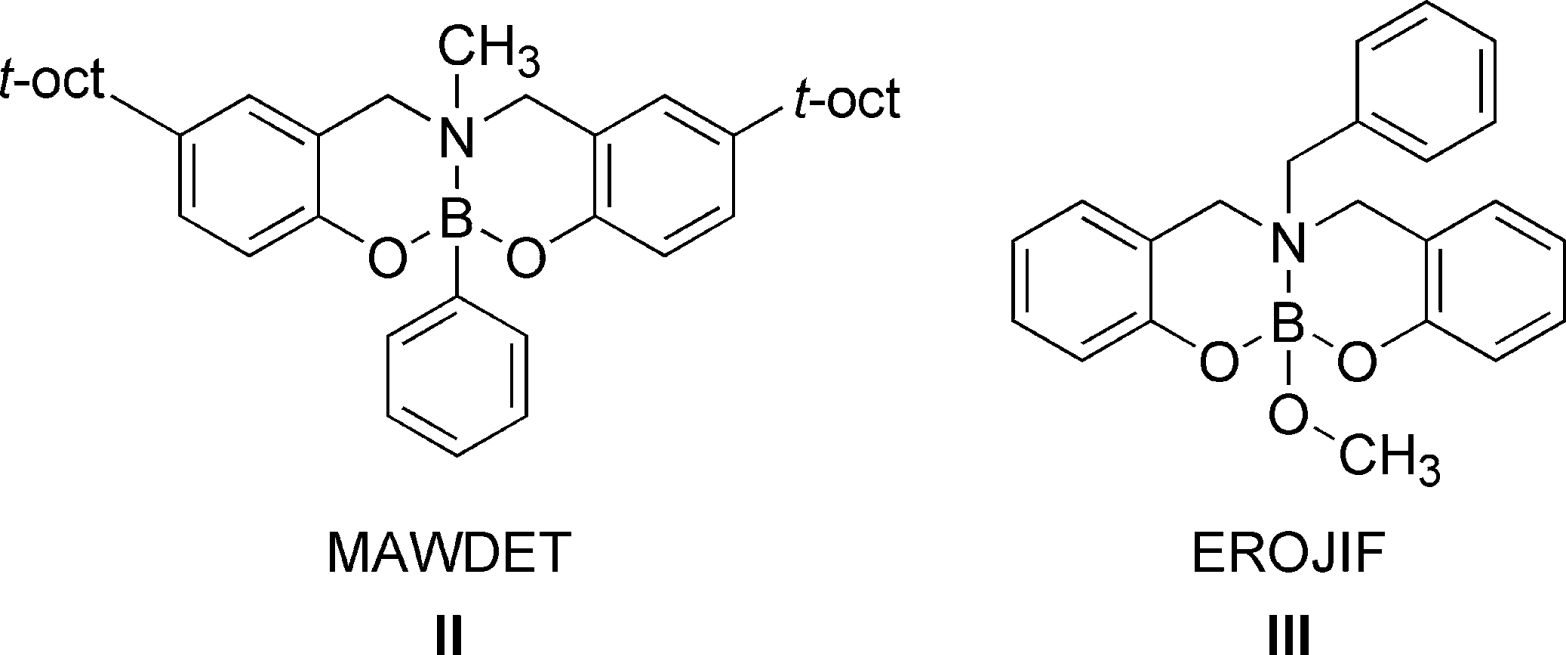
Oxaza­borocine compounds structurally related to the title compound.

A survey of the Cambridge Structural Database (CSD Version 5.38; Groom *et al.*, 2016 ▸) showed a few reported examples of dioxaza­borocines (Geng & Wu, 2011 ▸; Gawdzik *et al.*, 2009 ▸; Zhu *et al.*, 2006 ▸; Thadani *et al.*, 2001 ▸; Woodgate *et al.*, 2000 ▸; Woodgate *et al.*, 1999 ▸). Specifically, two members of this selected group are structurally related to the title compound: **II** (MAWDET; Woodgate *et al.*, 1999 ▸) and the recently described compound **III** (EROJIF; Geng & Wu, 2011 ▸) (Fig. 4 ▸).

Table 2 ▸ summarizes relevant bond lengths and angles for **I** compared with those observed in **II** and **III.** The intra­molecular N—B bond lengths can vary, depending on the substituent groups to boron and nitro­gen atoms. In particular, the covalent N1—B1 bond distance for **I** [1.674 (4) Å] is in the range observed for **III** and **II** [1.641 (2)–1.674 (5) Å]. The N—B bond distance for **III** is shorter than that in **II**, quite probably due to the extra oxygen atom bonded to the boron atom (from the –OCH_3_ group).

**Figure 5:**
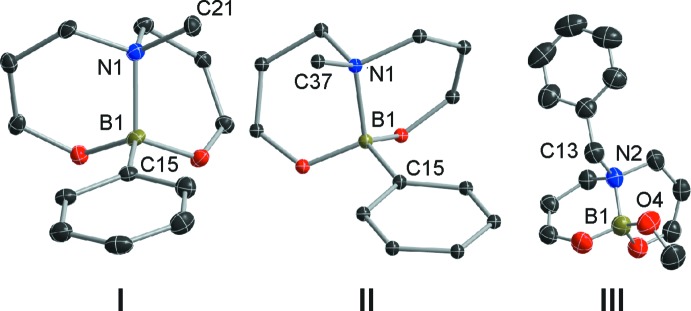
Comparison of the bonding environment at boron in **I** (title compound), **II** (MAWDET) and **III** (EROJIF). For clarity, only atoms closely involved in the N→B dative bonds are shown.

The crystal structure of **I** shows that the phenyl group at the boron atom and the *N*-pyridin-2-ylmethyl substituent adopts a *cis* conformation around the N→B dative bond, in total agreement with that reported for **II** and **III**. The C21—N1—B1—C15 torsion angle assumes a value of 57.8 (3)°. Analysis of the structural data for **II** showed the corresponding torsion angle (C37—N1—B1—C15) is 56.71°. In compound **III**, the corresponding angle (C13—N2—B1—O4) is 62.34°. These two examples display a *cis* geometry around the intra­molecular N—B bond, in concordance with compound **I** (Fig. 5 ▸).

We have performed an analysis of the experimental data of compounds **I-**-**III** and calculated the tetra­hedral character (THC_DA_) at the boron atom (Höpfl *et al.*, 1999 ▸), making use of the values of the six angles around the boron atom (θ_1_–θ_6_). The quite high value of 82.8% for **I** is in the range observed for compounds **II** and **III**. Altogether, this parameter and the measured N—B bond lengths can be considered a clear indication of *sp* ^3^-hybridization of the boron atom and of a resident negative charge (Sarina *et al.*, 2015 ▸). Therefore, we confirm that compound **I** adopts a zwitterionic form with a significant intra­molecular N→B dative bond.

**Figure 6:**
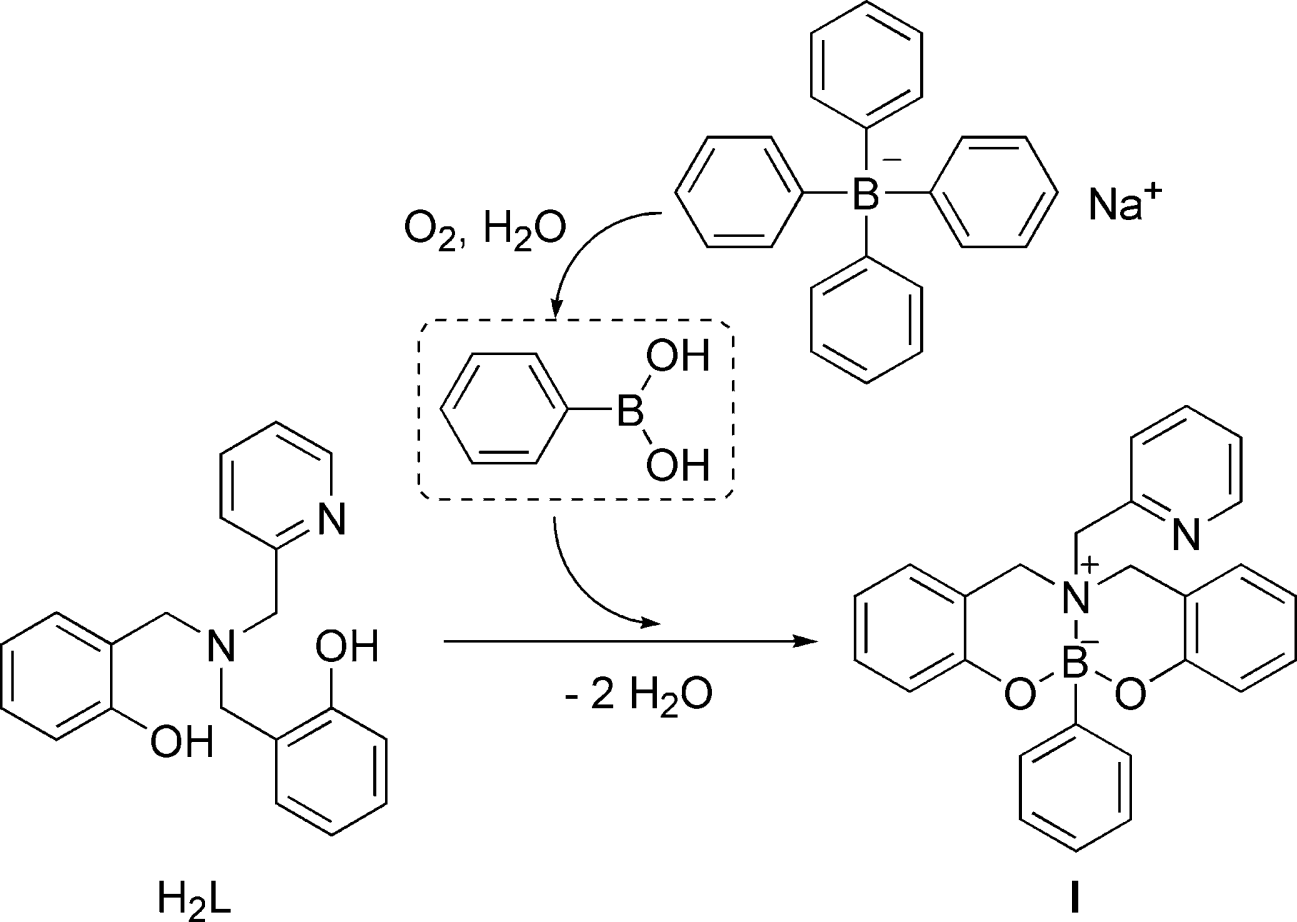
Synthesis of the title compound.

Based on previous observations (Barnes *et al.*, 1998 ▸), we hypothesize that employing an aqueous solution of NaBPh_4_ led to the unexpected isolation of **I**. It is well known that NaBPh_4_ in the presence of oxygen leads to the production of phenyl­boronic acid PhB(OH)_2_ and phenol. Then, the *in situ* generated phenyl­boronic acid (derived in turn from an excess of NaBPh_4_) is capable of reacting with the tripodal ligand H_2_ *L*, leading to the formation of compound **I** (Fig. 6 ▸).

Inspection of the reaction conditions already reported by Woodgate *et al.* (1999 ▸) indicates that compound **II** was obtained by reaction of phenyl­boronic acid and the corres­ponding tertiary amine. In turn, the authors reported that compound **III** was obtained unintentionally when using salicyl­aldehyde benzyl­amine and boron compounds (Geng *et al.*, 2011 ▸). We hypothesize that, in the case of **I**, the use of NaBPh_4_ determined the course of the reaction, leading to the formation of the zwitterionic heterocycle in the described reaction conditions.

## Synthesis and crystallization   

H_2_ *L* (0.064 g; 0.2 mmol) was dissolved in methanol (4 mL), then solid manganese(III) acetate dihydrate (0.052 g; 0.2 mmol) was added. Immediately after, an excess of NaBPh_4_ (0.2065 g, 0.60 mmol) in 2 mL of methanol/water was added to the reaction flask. The resulting dark-brown solution was sonicated at 313 K for 15 min and then stirred at reflux for additional 16 h (overnight). After cooling, the obtained precipitate was collected by filtration, washed with diethyl ether and dried *in vacuo*. Recrystallization from methanol gave colourless crystals of **I** suitable for X-ray diffraction. Yield: 21%. IR spectrum: ν(cm^−1^): 3043, 1630 (C=N), 1626, 1608 (C=C aromatic), 1462 (*br*, B—O), 1273, 1248, 1200, 1050 (C—O), 1002 (B—N), 702.

## Refinement   

Crystal data, data collection and structure refinement details are summarized in Table 3 ▸. H atoms were placed at calculated positions, with *d*(C—H) = 0.95−0.99 Å and *U*_iso_(H) = 1.2*U*_eq_(C).

## Supplementary Material

Crystal structure: contains datablock(s) global, I. DOI: 10.1107/S2056989017016553/rz5224sup1.cif

Structure factors: contains datablock(s) I. DOI: 10.1107/S2056989017016553/rz5224Isup2.hkl

Supporting information file. DOI: 10.1107/S2056989017016553/rz5224Isup3.cml

CCDC reference: 1586032

Additional supporting information:  crystallographic information; 3D view; checkCIF report

## Figures and Tables

**Table 1:** Hydrogen-bond geometry (Å, °)

*D*—H⋯*A*	*D*—H	H⋯*A*	*D*⋯*A*	*D*—H⋯*A*
C2—H2⋯O1^i^	0.95	2.57	3.503 (3)	168
C24—H24⋯O2^ii^	0.95	2.50	3.185 (3)	129

Symmetry codes: (i) 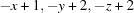; (ii) 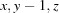.

**Table 2:** Structural data and calculated tetra­hedral character THC_DA_ (Å, °) for compounds **I**–**III**

	Compound **I**	**II** (MAWDET)^*a*^	**III** (EROJIF)^*b*^
*Bond lengths*			
B—N	1.674 (4) (B1—N1)	1.674 (5) (B1—N1)	1.641 (2) (B1—N2)
B—O	1.471 (3) (B1—O2)	1.443 (4) (B1—O2)	1.443 (2) (B1—O3)
B—O	1.446 (3) (B1—O1)	1.454 (4) (B1—O1)	1.463 (2) (B1—O5)
B—C	1.602 (4) (B1—C15)	1.608 (5) (B1—C15)	1.425 (2) (B1—O4)
*Angles*			
θ_1_	113.0 (2) (C15—B1—O2)	110.3 (3) (C15—B—O2)	113.34 (15) (O4—B1—O3)
θ_2_	109.5 (2) (C15—B1—O1)	115.5 (3) (C15—B—O1)	114.47 (16) (O4—B1—O5)
θ_3_	109.0 (2) (O2—B1—O1)	109.5 (3) (O2—B—O1)	108.60 (15) (O3—B1—O5)
θ_4_	113.5 (2) (N1—B1—C15)	110.0 (3) (N1—B—C15)	105.64 (14) (N2—B1—O4)
θ_5_	104.5 (2) (N1—B1—O2)	106.7 (3) (N1—B—O2)	108.45 (14) (N2—B1—O3)
θ_6_	107.1 (2) (N1—B1—O1)	104.4 (3) (N1—B—O1)	105.89 (13) (N2—B1—O5)
THC_DA_^*c*^	82.8	83.1	79.7

(*a*) Woodgate *et al.* (1999 ▸); (*b*) Geng *et al.* (2011 ▸); (*c*) Höpfl *et al.* (1999 ▸).

**Table 3:** Experimental details

Crystal data	
Chemical formula	C_26_H_23_BN_2_O_2_
*M*_r_	406.27
Crystal system, space group	Triclinic, *P* 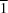
Temperature (K)	100
*a*, *b*, *c* (Å)	8.8803 (7), 10.0871 (8), 11.7586 (10)
α, β, γ (°)	97.298 (2), 98.464 (2), 98.234 (2)
*V* (Å^3^)	1019.21 (14)
*Z*	2
Radiation type	Mo *K*α
μ (mm^−1^)	0.08
Crystal size (mm)	0.22 × 0.16 × 0.12

Data collection	
Diffractometer	Bruker APEXII CCD
Absorption correction	Gaussian (*XPREP* and *SADABS*; Bruker, 2008 ▸)
*T*_min_, *T*_max_	0.780, 0.875
No. of measured, independent and observed [*I* > 2σ(*I*)] reflections	8344, 4004, 2161
*R*_int_	0.088
(sin θ/λ)_max_ (Å^−1^)	0.617

Refinement	
*R*[*F* ^2^ > 2σ(*F* ^2^)], *wR*(*F* ^2^), *S*	0.059, 0.115, 0.92
No. of reflections	4004
No. of parameters	280
H-atom treatment	H-atom parameters constrained
Δρ_max_, Δρ_min_ (e Å^−3^)	0.25, −0.31

Computer programs: *APEX2* and *SAINT* (Bruker, 2008 ▸), *SHELXS97* and *SHELXTL* (Sheldrick, 2008 ▸), *SHELXL2016* (Sheldrick, 2015 ▸) and *DIAMOND* (Brandenburg, 2012 ▸).

## supplementary crystallographic information

### Crystal data

**Table:**

C_26_H_23_BN_2_O_2_	*Z* = 2
*M**_r_* = 406.27	*F*(000) = 428
Triclinic, *P*1	*D*_x_ = 1.324 Mg m^−^^3^
Hall symbol: -P 1	Mo *K*α radiation, λ = 0.71073 Å
*a* = 8.8803 (7) Å	Cell parameters from 5951 reflections
*b* = 10.0871 (8) Å	θ = 2.4–28.2°
*c* = 11.7586 (10) Å	µ = 0.08 mm^−^^1^
α = 97.298 (2)°	*T* = 100 K
β = 98.464 (2)°	Prism, colourless
γ = 98.234 (2)°	0.22 × 0.16 × 0.12 mm
*V* = 1019.21 (14) Å^3^	

### Data collection

**Table:**

Bruker APEXII CCD diffractometer	2161 reflections with *I* > 2σ(*I*)
Graphite monochromator	*R*_int_ = 0.088
φ and ω scans	θ_max_ = 26.0°, θ_min_ = 2.4°
Absorption correction: gaussian (XPREP and SADABS; Bruker, 2008)	*h* = −10→10
*T*_min_ = 0.780, *T*_max_ = 0.875	*k* = −12→10
8344 measured reflections	*l* = −14→14
4004 independent reflections	

### Refinement

**Table:**

Refinement on *F*^2^	Primary atom site location: structure-invariant direct methods
Least-squares matrix: full	Secondary atom site location: difference Fourier map
*R*[*F*^2^ > 2σ(*F*^2^)] = 0.059	Hydrogen site location: inferred from neighbouring sites
*wR*(*F*^2^) = 0.115	H-atom parameters constrained
*S* = 0.92	*w* = 1/[σ^2^(*F*_o_^2^) + (0.0336*P*)^2^] where *P* = (*F*_o_^2^ + 2*F*_c_^2^)/3
4004 reflections	(Δ/σ)_max_ < 0.001
280 parameters	Δρ_max_ = 0.25 e Å^−^^3^
0 restraints	Δρ_min_ = −0.31 e Å^−^^3^

### Special details

**Table:**

Geometry. All esds (except the esd in the dihedral angle between two l.s. planes) are estimated using the full covariance matrix. The cell esds are taken into account individually in the estimation of esds in distances, angles and torsion angles; correlations between esds in cell parameters are only used when they are defined by crystal symmetry. An approximate (isotropic) treatment of cell esds is used for estimating esds involving l.s. planes.
Refinement. Refinement of F^2^ against ALL reflections. The weighted R-factor wR and goodness of fit S are based on F^2^, conventional R-factors R are based on F, with F set to zero for negative F^2^. The threshold expression of F^2^ > 2sigma(F^2^) is used only for calculating R-factors(gt) etc. and is not relevant to the choice of reflections for refinement. R-factors based on F^2^ are statistically about twice as large as those based on F, and R- factors based on ALL data will be even larger.

### Fractional atomic coordinates and isotropic or equivalent isotropic displacement parameters (Å^2^)

**Table:**

	*x*	*y*	*z*	*U*_iso_*/*U*_eq_	
O2	0.33571 (19)	0.80339 (16)	0.66654 (14)	0.0162 (4)	
C7	0.2240 (3)	0.5768 (2)	0.7698 (2)	0.0154 (6)	
H7A	0.186984	0.481569	0.777707	0.018*	
H7B	0.15142	0.602455	0.707238	0.018*	
O1	0.45343 (19)	0.81457 (16)	0.86313 (14)	0.0155 (4)	
N1	0.3811 (2)	0.58730 (19)	0.73635 (17)	0.0142 (5)	
N2	0.3294 (3)	0.3306 (2)	0.87553 (18)	0.0228 (6)	
C1	0.3311 (3)	0.7832 (2)	0.9193 (2)	0.0141 (6)	
C15	0.6210 (3)	0.7798 (2)	0.7133 (2)	0.0145 (6)	
C6	0.2247 (3)	0.6666 (2)	0.8822 (2)	0.0143 (6)	
C8	0.2760 (3)	0.7354 (2)	0.5582 (2)	0.0149 (6)	
C22	0.4325 (3)	0.3686 (2)	0.8075 (2)	0.0164 (6)	
C20	0.6479 (3)	0.8046 (2)	0.6034 (2)	0.0168 (6)	
H20	0.562356	0.799166	0.543102	0.02*	
C14	0.3653 (3)	0.5215 (2)	0.6124 (2)	0.0159 (6)	
H14A	0.469245	0.515167	0.593371	0.019*	
H14B	0.307445	0.428216	0.603349	0.019*	
C13	0.2833 (3)	0.5988 (2)	0.5288 (2)	0.0154 (6)	
C2	0.3191 (3)	0.8716 (3)	1.0178 (2)	0.0184 (6)	
H2	0.391788	0.952737	1.042134	0.022*	
C19	0.7968 (3)	0.8371 (3)	0.5794 (2)	0.0218 (7)	
H19	0.811445	0.851654	0.503233	0.026*	
C3	0.2016 (3)	0.8410 (3)	1.0797 (2)	0.0209 (7)	
H3	0.192493	0.901899	1.145982	0.025*	
C26	0.4860 (3)	0.2759 (2)	0.7329 (2)	0.0183 (6)	
H26	0.56079	0.305733	0.687579	0.022*	
C5	0.1087 (3)	0.6353 (3)	0.9472 (2)	0.0195 (6)	
H5	0.036794	0.553633	0.923746	0.023*	
C9	0.2030 (3)	0.8050 (3)	0.4773 (2)	0.0190 (7)	
H9	0.198548	0.898346	0.497886	0.023*	
C16	0.7521 (3)	0.7913 (2)	0.7980 (2)	0.0197 (7)	
H16	0.738836	0.77471	0.873908	0.024*	
C12	0.2147 (3)	0.5337 (3)	0.4180 (2)	0.0183 (6)	
H12	0.216587	0.439785	0.397566	0.022*	
C4	0.0970 (3)	0.7221 (3)	1.0458 (2)	0.0226 (7)	
H4	0.017669	0.699995	1.089651	0.027*	
C25	0.4296 (3)	0.1387 (3)	0.7247 (2)	0.0222 (7)	
H25	0.464256	0.073442	0.673508	0.027*	
C17	0.9002 (3)	0.8257 (3)	0.7761 (2)	0.0217 (7)	
H17	0.986288	0.834068	0.836496	0.026*	
C10	0.1376 (3)	0.7395 (3)	0.3682 (2)	0.0223 (7)	
H10	0.087663	0.787534	0.313513	0.027*	
C21	0.4862 (3)	0.5193 (2)	0.8156 (2)	0.0168 (6)	
H21A	0.591196	0.534146	0.795601	0.02*	
H21B	0.492924	0.562668	0.896988	0.02*	
C24	0.3225 (3)	0.0994 (3)	0.7923 (2)	0.0204 (7)	
H24	0.2805	0.006461	0.788101	0.024*	
C11	0.1438 (3)	0.6029 (3)	0.3371 (2)	0.0224 (7)	
H11	0.099895	0.557761	0.260997	0.027*	
C18	0.9221 (3)	0.8480 (2)	0.6656 (2)	0.0226 (7)	
H18	1.023545	0.870859	0.649451	0.027*	
C23	0.2776 (3)	0.1973 (3)	0.8658 (2)	0.0239 (7)	
H23	0.204908	0.168795	0.913098	0.029*	
B1	0.4511 (3)	0.7520 (3)	0.7450 (3)	0.0153 (7)	

### Atomic displacement parameters (Å^2^)

**Table:**

	*U*^11^	*U*^22^	*U*^33^	*U*^12^	*U*^13^	*U*^23^
O2	0.0199 (11)	0.0149 (10)	0.0135 (10)	0.0035 (8)	0.0013 (8)	0.0025 (8)
C7	0.0131 (15)	0.0146 (15)	0.0167 (15)	−0.0010 (12)	0.0014 (12)	0.0005 (12)
O1	0.0169 (10)	0.0147 (10)	0.0132 (10)	−0.0016 (8)	0.0041 (8)	−0.0008 (8)
N1	0.0138 (12)	0.0135 (12)	0.0141 (12)	0.0002 (9)	0.0017 (10)	0.0012 (9)
N2	0.0325 (15)	0.0179 (14)	0.0204 (14)	0.0036 (11)	0.0096 (12)	0.0061 (11)
C1	0.0124 (15)	0.0148 (15)	0.0151 (15)	0.0013 (12)	0.0020 (12)	0.0038 (12)
C15	0.0188 (15)	0.0087 (14)	0.0155 (15)	0.0021 (11)	0.0023 (12)	0.0002 (11)
C6	0.0158 (15)	0.0140 (15)	0.0131 (14)	0.0009 (12)	0.0021 (12)	0.0043 (12)
C8	0.0127 (14)	0.0195 (15)	0.0113 (14)	−0.0002 (12)	0.0030 (12)	0.0000 (12)
C22	0.0192 (16)	0.0147 (15)	0.0143 (15)	0.0030 (12)	−0.0025 (13)	0.0046 (12)
C20	0.0165 (16)	0.0133 (15)	0.0195 (16)	−0.0007 (12)	0.0029 (13)	0.0018 (12)
C14	0.0213 (16)	0.0119 (14)	0.0131 (14)	0.0016 (12)	0.0041 (12)	−0.0036 (11)
C13	0.0164 (15)	0.0186 (15)	0.0120 (14)	0.0025 (12)	0.0037 (12)	0.0043 (12)
C2	0.0231 (17)	0.0171 (15)	0.0140 (15)	0.0029 (13)	0.0009 (13)	0.0013 (12)
C19	0.0253 (17)	0.0179 (15)	0.0210 (16)	−0.0003 (13)	0.0055 (14)	0.0015 (13)
C3	0.0284 (17)	0.0200 (16)	0.0146 (15)	0.0036 (13)	0.0080 (13)	−0.0008 (12)
C26	0.0182 (16)	0.0173 (16)	0.0209 (16)	0.0019 (12)	0.0071 (13)	0.0052 (12)
C5	0.0200 (16)	0.0170 (15)	0.0196 (16)	−0.0012 (12)	0.0022 (13)	0.0018 (12)
C9	0.0208 (16)	0.0172 (15)	0.0208 (16)	0.0049 (12)	0.0065 (13)	0.0043 (13)
C16	0.0224 (17)	0.0198 (16)	0.0170 (16)	0.0034 (13)	0.0049 (13)	0.0021 (12)
C12	0.0174 (15)	0.0202 (15)	0.0174 (16)	0.0014 (12)	0.0049 (13)	0.0029 (12)
C4	0.0281 (18)	0.0237 (17)	0.0183 (16)	0.0047 (14)	0.0095 (14)	0.0051 (13)
C25	0.0270 (17)	0.0165 (16)	0.0229 (16)	0.0074 (13)	0.0031 (14)	−0.0005 (12)
C17	0.0152 (16)	0.0222 (16)	0.0258 (17)	0.0028 (13)	0.0003 (13)	−0.0001 (13)
C10	0.0196 (16)	0.0322 (17)	0.0162 (16)	0.0085 (13)	0.0012 (13)	0.0056 (13)
C21	0.0193 (15)	0.0170 (15)	0.0137 (15)	0.0050 (12)	−0.0010 (12)	0.0028 (12)
C24	0.0257 (17)	0.0131 (15)	0.0217 (16)	0.0003 (12)	0.0027 (13)	0.0049 (12)
C11	0.0181 (16)	0.0306 (17)	0.0155 (15)	−0.0012 (13)	0.0013 (13)	0.0001 (13)
C18	0.0218 (17)	0.0160 (16)	0.0307 (18)	0.0002 (13)	0.0111 (15)	0.0019 (13)
C23	0.0266 (18)	0.0220 (17)	0.0259 (17)	0.0037 (13)	0.0094 (14)	0.0089 (13)
B1	0.0192 (18)	0.0107 (16)	0.0141 (17)	0.0007 (13)	0.0022 (14)	−0.0022 (13)

### Geometric parameters (Å, º)

**Table:**

O2—C8	1.360 (3)	C2—H2	0.95
O2—B1	1.471 (3)	C19—C18	1.372 (4)
C7—N1	1.497 (3)	C19—H19	0.95
C7—C6	1.504 (3)	C3—C4	1.382 (4)
C7—H7A	0.99	C3—H3	0.95
C7—H7B	0.99	C26—C25	1.389 (4)
O1—C1	1.372 (3)	C26—H26	0.95
O1—B1	1.446 (3)	C5—C4	1.387 (3)
N1—C14	1.501 (3)	C5—H5	0.95
N1—C21	1.517 (3)	C9—C10	1.370 (4)
N1—B1	1.674 (4)	C9—H9	0.95
N2—C23	1.343 (3)	C16—C17	1.381 (4)
N2—C22	1.348 (3)	C16—H16	0.95
C1—C6	1.378 (3)	C12—C11	1.381 (3)
C1—C2	1.396 (3)	C12—H12	0.95
C15—C20	1.396 (3)	C4—H4	0.95
C15—C16	1.396 (3)	C25—C24	1.374 (3)
C15—B1	1.602 (4)	C25—H25	0.95
C6—C5	1.395 (3)	C17—C18	1.383 (3)
C8—C13	1.391 (3)	C17—H17	0.95
C8—C9	1.391 (3)	C10—C11	1.391 (4)
C22—C26	1.383 (3)	C10—H10	0.95
C22—C21	1.513 (3)	C21—H21A	0.99
C20—C19	1.394 (4)	C21—H21B	0.99
C20—H20	0.95	C24—C23	1.371 (3)
C14—C13	1.501 (3)	C24—H24	0.95
C14—H14A	0.99	C11—H11	0.95
C14—H14B	0.99	C18—H18	0.95
C13—C12	1.391 (3)	C23—H23	0.95
C2—C3	1.380 (3)		
			
C8—O2—B1	121.39 (18)	C22—C26—C25	119.4 (2)
N1—C7—C6	111.9 (2)	C22—C26—H26	120.3
N1—C7—H7A	109.2	C25—C26—H26	120.3
C6—C7—H7A	109.2	C4—C5—C6	120.8 (3)
N1—C7—H7B	109.2	C4—C5—H5	119.6
C6—C7—H7B	109.2	C6—C5—H5	119.6
H7A—C7—H7B	107.9	C10—C9—C8	120.3 (2)
C1—O1—B1	120.8 (2)	C10—C9—H9	119.9
C7—N1—C14	108.67 (19)	C8—C9—H9	119.9
C7—N1—C21	110.68 (18)	C17—C16—C15	122.9 (2)
C14—N1—C21	110.23 (16)	C17—C16—H16	118.6
C7—N1—B1	107.64 (16)	C15—C16—H16	118.6
C14—N1—B1	108.46 (18)	C11—C12—C13	121.2 (2)
C21—N1—B1	111.07 (19)	C11—C12—H12	119.4
C23—N2—C22	116.7 (2)	C13—C12—H12	119.4
O1—C1—C6	121.5 (2)	C3—C4—C5	119.5 (3)
O1—C1—C2	118.0 (2)	C3—C4—H4	120.3
C6—C1—C2	120.5 (2)	C5—C4—H4	120.3
C20—C15—C16	115.9 (2)	C24—C25—C26	118.6 (2)
C20—C15—B1	122.7 (2)	C24—C25—H25	120.7
C16—C15—B1	121.2 (2)	C26—C25—H25	120.7
C1—C6—C5	119.0 (2)	C16—C17—C18	119.5 (3)
C1—C6—C7	121.8 (2)	C16—C17—H17	120.3
C5—C6—C7	119.1 (2)	C18—C17—H17	120.3
O2—C8—C13	121.4 (2)	C9—C10—C11	120.4 (2)
O2—C8—C9	118.4 (2)	C9—C10—H10	119.8
C13—C8—C9	120.3 (2)	C11—C10—H10	119.8
N2—C22—C26	122.3 (2)	C22—C21—N1	113.5 (2)
N2—C22—C21	116.5 (2)	C22—C21—H21A	108.9
C26—C22—C21	121.2 (2)	N1—C21—H21A	108.9
C19—C20—C15	121.9 (3)	C22—C21—H21B	108.9
C19—C20—H20	119	N1—C21—H21B	108.9
C15—C20—H20	119	H21A—C21—H21B	107.7
C13—C14—N1	112.13 (17)	C23—C24—C25	118.5 (3)
C13—C14—H14A	109.2	C23—C24—H24	120.8
N1—C14—H14A	109.2	C25—C24—H24	120.8
C13—C14—H14B	109.2	C12—C11—C10	119.2 (3)
N1—C14—H14B	109.2	C12—C11—H11	120.4
H14A—C14—H14B	107.9	C10—C11—H11	120.4
C8—C13—C12	118.6 (2)	C19—C18—C17	119.7 (3)
C8—C13—C14	121.8 (2)	C19—C18—H18	120.1
C12—C13—C14	119.6 (2)	C17—C18—H18	120.1
C3—C2—C1	119.9 (3)	N2—C23—C24	124.5 (3)
C3—C2—H2	120.1	N2—C23—H23	117.8
C1—C2—H2	120.1	C24—C23—H23	117.8
C18—C19—C20	120.1 (3)	O1—B1—O2	108.95 (17)
C18—C19—H19	120	O1—B1—C15	109.5 (2)
C20—C19—H19	120	O2—B1—C15	113.0 (2)
C2—C3—C4	120.3 (2)	O1—B1—N1	107.1 (2)
C2—C3—H3	119.8	O2—B1—N1	104.5 (2)
C4—C3—H3	119.8	C15—B1—N1	113.46 (17)

### Hydrogen-bond geometry (Å, º)

**Table:**

*D*—H···*A*	*D*—H	H···*A*	*D*···*A*	*D*—H···*A*
C2—H2···O1^i^	0.95	2.57	3.503 (3)	168
C24—H24···O2^ii^	0.95	2.50	3.185 (3)	129

Symmetry codes: (i) −*x*+1, −*y*+2, −*z*+2; (ii) *x*, *y*−1, *z*.
